# Predictive Value of Preoperative Fibrinogen and Albumin Score (FA Score) for Prognosis and Chemotherapeutic Efficacy in Resected Colorectal Cancer: A Retrospective Cohort Study

**DOI:** 10.7150/jca.100674

**Published:** 2024-09-23

**Authors:** Bang An, Tong Liu, Xiao Li

**Affiliations:** 1 Department of Gastroenterology, Shandong Provincial Hospital Affiliated to Shandong First Medical University, Jinan 250021, Shandong Province, China.; 2 Department of Cardiology, Central Hospital Affiliated to Shandong First Medical University, Jinan Central Hospital, Jinan 250013, Shandong Province, China.

**Keywords:** colorectal cancer, fibrinogen, albumin, prognosis, chemotherapeutic efficacy

## Abstract

**Background:** Limited research elucidated the role of preoperative fibrinogen and albumin (FA) score in colorectal cancer (CRC). We aimed to clarify the predictive value of FA score for prognosis and chemotherapeutic efficacy in CRC patients who underwent curative resection.

**Materials and Methods:** Patients' clinicopathological parameters of 735 cases of resected CRC were recruited retrospectively. Optimal cut-off values of the preoperative plasma fibrinogen (F) and albumin (A) were confirmed by receiver operating characteristic (ROC) curves. Patients were categorized into three groups based on the FA score, and were further divided into a chemotherapy group and a non-chemotherapy group. Correlations between FA score and clinicopathological features, as well as overall survival (OS), cancer-specific survival (CSS) and disease-free survival (DFS) were assessed with Kaplan-Meier (KM) survival method, univariate and multivariate Cox proportional hazard models, and subgroup analyses.

**Results:** The Kaplan-Meier survival curves revealed that higher FA score could predict poorer OS and CSS (P<0.001). Multivariate analyses revealed that FA score was an independent prognostic factor for OS (P=0.037). In addition, subgroup analyses based on the histological feature and primary tumor location showed that elevated FA score was significantly associated with worse OS, CSS and DFS (all, P<0.05) in patients with non-mucinous colorectal adenocarcinoma and rectal cancer (RECC). Subgroup analyses based on the TNM stage showed that elevated FA score was significantly associated with worse OS, CSS (all, P<0.05) in patients with TNM stage II tumors. Furthermore, chemotherapy could benefit the OS and CSS in TNM stage III CRC patients with FA score 1 and 2 (all, P<0.05).

**Conclusion:** The preoperative FA score is an independent prognostic factor for CRC patients who underwent curative resection and may help predict the responses to chemotherapy in clinical practice. FA score may serve as a complementary to the TNM staging system to identify high-risk patients.

## Introduction

Colorectal cancer (CRC) is the third most common malignancies and the second leading cause of cancer-related death worldwide 1. CRC also has the fifth highest incidence and the fifth highest mortality rate in China 2. For early-stage CRCs, curative surgery remains the mainstay treatment strategy; while for patients with stage III and high-risk stage II CRCs, adjuvant chemotherapy is strongly recommended 3, 4. Despite of the dramatic progress achieved in diagnosis, surgical procedures, neoadjuvant and adjuvant therapies in decades, a majority of CRC patients were diagnosed with metastasis and the 5-year survival rate remains relatively poor 5. Therefore, there is an urgent need to seek for novel, stable, effective and economical predictors to evaluate the survival time and chemotherapy efficacy after surgery.

Accumulating evidence indicates that cancer-related systemic inflammation 6, 7, hemostatic factors 8 and nutritional deficiencies 9 might facilitate the tumorigenesis and progression of various malignancies. Recently, several inflammation-based indicators have been demonstrated to be crucial for the aggressiveness and poor prognosis of CRC, including the neutrophil-lymphocyte ratio 10, the combined fibrinogen and neutrophil-lymphocyte-ratio (F-NLR) 11, the Glasgow Prognostic Score (GPS) 12 and the systemic immune-inflammation index (SII) 13. On the other hand, previous studies have indicated that elevated plasma fibrinogen level 14 and decreased serum albumin level 15 are associated with poor outcomes in patients with CRC.

Recent studies have emphasized that the FA score, combination of the fibrinogen (F) and albumin (A), was an effective predictor for the prognosis of various carcinomas, such as esophageal cancer 16, gastric cancer 17, non-small cell lung cancer 18, WHO Grade II/III Gliomas 19 and epithelial ovarian cancer 20. However, to the best of our knowledge, the predictive value of FA score for the prognosis and chemotherapeutic efficacy in CRC patients still needs to be fully elucidated. Thus, we decided to investigate the prognostic and predictive value of FA score in a cohort of Chinese CRC patients who underwent curative resection in the present study.

## Materials and Methods

### Patients

A total of 735 CRC patients who underwent radical resection at Shandong Provincial Hospital Affiliated to Shandong First Medical University between Jan 2009 and July 2016 were recruited retrospectively. Patients enrolled in the research met the following enrollment criteria: (1) first diagnosed and pathologically confirmed primary CRC; (2) radical surgery without preoperative neoadjuvant chemotherapy or radiotherapy; (3) complete resection without positive margins; (4) intact medical data and follow-up record (more than 2 months). Patients were excluded from the study if they: (1) had preexisting liver diseases, chronic renal failure, hematological disorders, autoimmune diseases, recent infection, or other malignancies; (2) received immunosuppressive, anti-inflammatory, or anticoagulation treatment; (3) had a recent history of venous thrombosis or blood transfusion.

### Data collection

Patients' clinical baseline features were collected from medical records: age, gender, smoking, drinking, morphology, histological type, differentiation, primary tumor location, tumor size, venous invasion, perineural invasion (PNI), tumor deposits (TDs), N stage, TNM stage, chemotherapy treatment, preoperative plasma fibrinogen, serum albumin, carcinoembryonic antigen (CEA) and cancer antigen 19-9 (CA19-9). The TNM stage was classified by the seventh edition of the American Joint Committee on Cancer (AJCC) staging manual 21.

### FA score measurement

**Figure 1:**
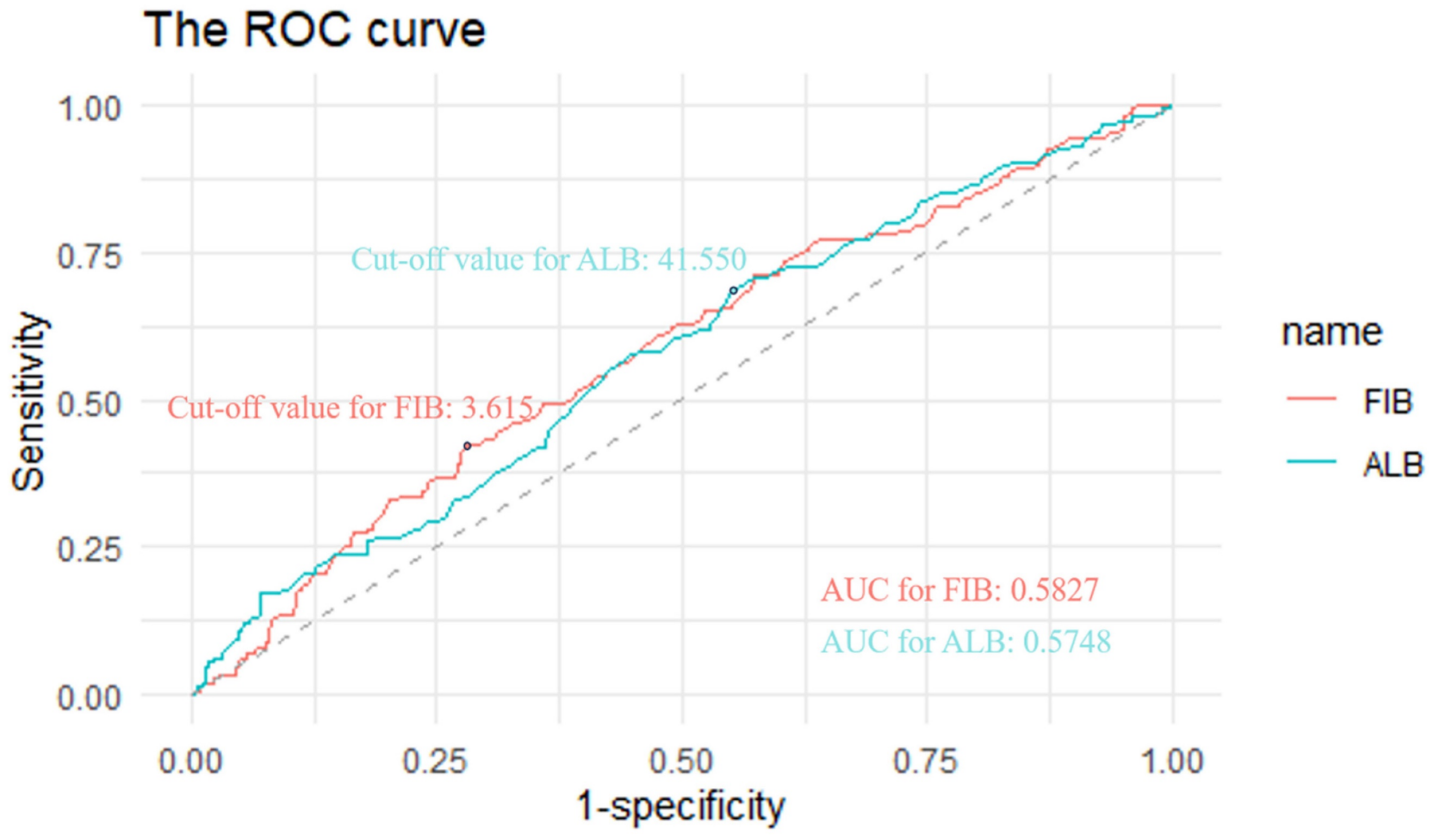
ROC curves to assess the predictive value of plasma fibrinogen and albumin. **Notes:** The cut-off values were 3.615 for fibrinogen and 41.550 g/L for albumin, respectively. The AUC values were 0.5827 for fibrinogen and 0.5748 for albumin, respectively. **Abbreviations:** FIB, fibrinogen; ALB, albumin; ROC, receiver operating characteristic. AUC, area under the curve.

Preoperative plasma fibrinogen and serum albumin levels were extracted to evaluate the FA score. The optimal cut-off values of fibrinogen and albumin were determined by the receiver operating characteristic (ROC) analysis based on the maximization of the Youden index. The cut-off values were 3.615 for fibrinogen and 41.550 g/L for albumin, respectively (Figure 1). The area under the curve (AUC) values were 0.5827 for fibrinogen and 0.5748 for albumin, respectively (Figure 1). The FA score was defined as follows: patients with an elevated fibrinogen and a decreased albumin were allocated a score of 2, those with only one of these abnormalities were assigned a score of 1, and those with neither of them were classified as a score of 0.

### Follow up

The overall survival (OS), cancer-specific survival (CSS) and disease-free survival (DFS) were selected as primary endpoints. The OS was measured from the date of surgery to the date of death. The CSS was defined as the time between the date of surgery and the date of cancer-related death. The DFS was defined as the interval between the date of surgery and the time of progression or relapse or the end of life. The median duration of follow-up was 30.47months (range: 3-102 months).

### Statistical analysis

All analyses were performed using the R software version 4.1.1. The optimal cut-off values for F and A were calculated with ROC analysis. The relationships among the FA score and other clinicopathological parameters were compared by chi-square test (χ²) test or the Fisher's exact test. The survival outcomes were compared using the Kaplan-Meier method, the log-rank test, the univariate and multivariate Cox proportional hazards model, and subset analysis. In the univariate analysis, variables with a P-value <0.1 were chosen to construct the multivariate Cox proportional hazards regression model. A two-sided P<0.05 was considered statistically significant.

## Results

### Patients' baseline characteristics

As presented in Table 1, a total of 735 resected CRC patients (63.95% male and 36.05% female) were enrolled in the present research. Primary tumors were located at left colon, right colon and rectum in 21.50%, 15.51% and 62.99% of the patients, respectively. Of all the patients, 612 (83.27%) tumors were adenocarcinoma and the remaining 123 (16.73%) tumors were mucinous adenocarcinoma. Approximately 40.41% of the patients were older than 60 years old; 19.86% and 16.87% of the patients had a history of smoking and drinking, separately; 39.18% had smaller tumor size; and 80.0% received postoperative adjuvant chemotherapy. Pathological stage (TNM stage) was I/ II/ III in 9.66%, 39.18% and 51.16% of the patients, respectively. Patients with the expansive, infiltrative, ulcerative and complex morphological type accounted for 16.60%, 2.99%, 78.91% and 1.50%, respectively.

Patients were further divided into three groups based on the FA score, of which 165 patients had a FA score of 0, 399 patients had a FA score of 1 and the remaining 171 patients had a FA score of 2. The relationship between FA score and clinicopathological features was also shown in Table 1. The results showed no significant differences between FA score 0, 1 and 2 groups in smoking, drinking, morphology, histology, differentiation, venous invasion, perineural invasion, tumor deposits, T stage, N stage and chemotherapy (all, P > 0.05). However, significant differences were identified among different FA score groups in terms of gender (P = 0.0325), age (P < 0.0001), CEA (P = 0.0096), TNM stage (P = 0.0032), primary tumor location (P < 0.0001) and tumor size (P < 0.0001) (Table 1).

### Univariate and multivariate survival analyses

The results of univariate and multivariate analyses for OS were summarized in Table 2. By univariate analysis, following variables were found to be associated with worse OS in patients with CRC: older age, positive venous invasion, positive perineural invasion, positive tumor deposits, higher T stage, higher N stage and higher FA score (all, P < 0.1). Multivariate analysis was conducted by controlling for these cofounders. Results revealed that the age (P=0.036), T stage (P=0.028), N stage (P<0.001) and FA score (P=0.037) were independent prognostic factors for OS. In addition, a higher FA score was significantly associated with worse prognosis.

The results of univariate and multivariate analyses for CSS were presented in Table 3. The age (P=0.018), primary tumor location (P=0.033 for the comparation of RCC and LCC), venous invasion (P=0.002), perineural invasion (P=0.004), tumor deposits (P=0.004), T stage (P<0.001), N stage (P<0.001) and FA score (P=0.007) were associated with CSS in the univariate analysis. Multivariate analysis demonstrated that the age (P=0.033), primary tumor location (P=0.047 for the comparation of RCC and LCC), T stage (P=0.008), N stage (P<0.001) were independent prognostic factors for CSS.

The results of univariate and multivariate analyses for DFS were shown in Table 4. By univariate analysis, LCC, negative venous invasion, negative perineural invasion, absence of tumor deposits, lower T stage, lower N stage, non-chemotherapy and lower FA score were associated with better DFS in patients with CRC (all, P < 0.1). Multivariate analysis after controlling for these variables revealed that the primary tumor location, perineural invasion, tumor deposits, N stage were independent prognostic factors for DFS (all, P < 0.05).

### Kaplan-Meier survival analyses

**Figure 2:**
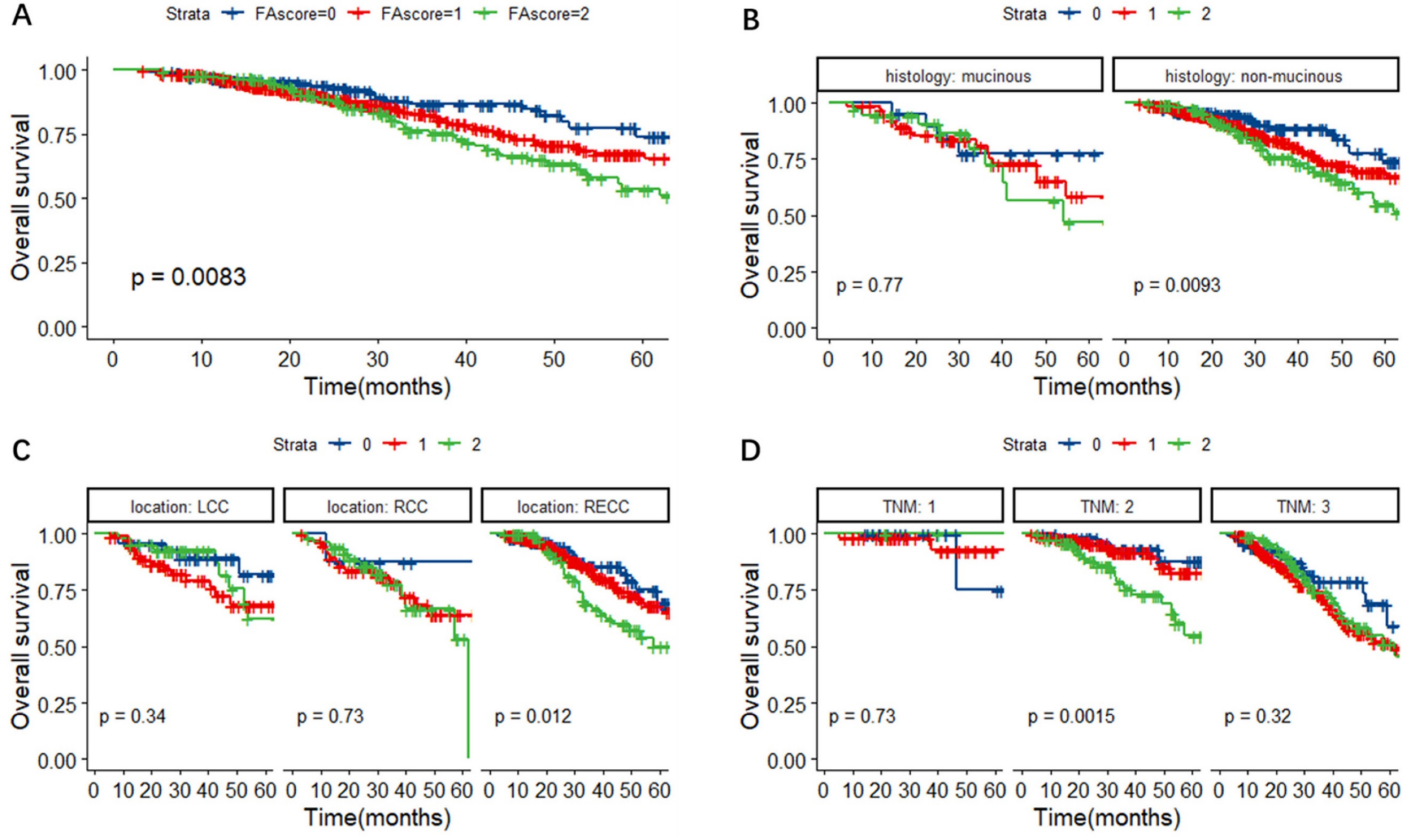
Kaplan-Meier survival curves for OS according to the FA score (A) and subgroup analysis based on histological features (B) and primary tumor location (C) and TNM stage (D). **Abbreviations:** FA score, combined fibrinogen and albumin; OS, overall survival.

In Figure 2A, the Kaplan-Meier survival curves for OS revealed that the FA score could predict the OS of CRC patients (P = 0.0083). Further subgroup analyses were performed to investigate the prognostic value of FA score in CRC patients with different histological types, primary tumor locations and TNM stages. As shown in Figure 2B-D, the FA score could indicate prognosis in CRC patients with non-mucinous adenocarcinoma (P = 0.0093), rectal cancer (RECC) (P = 0.012) and TNM stage II tumors (P = 0.0015).

**Figure 3:**
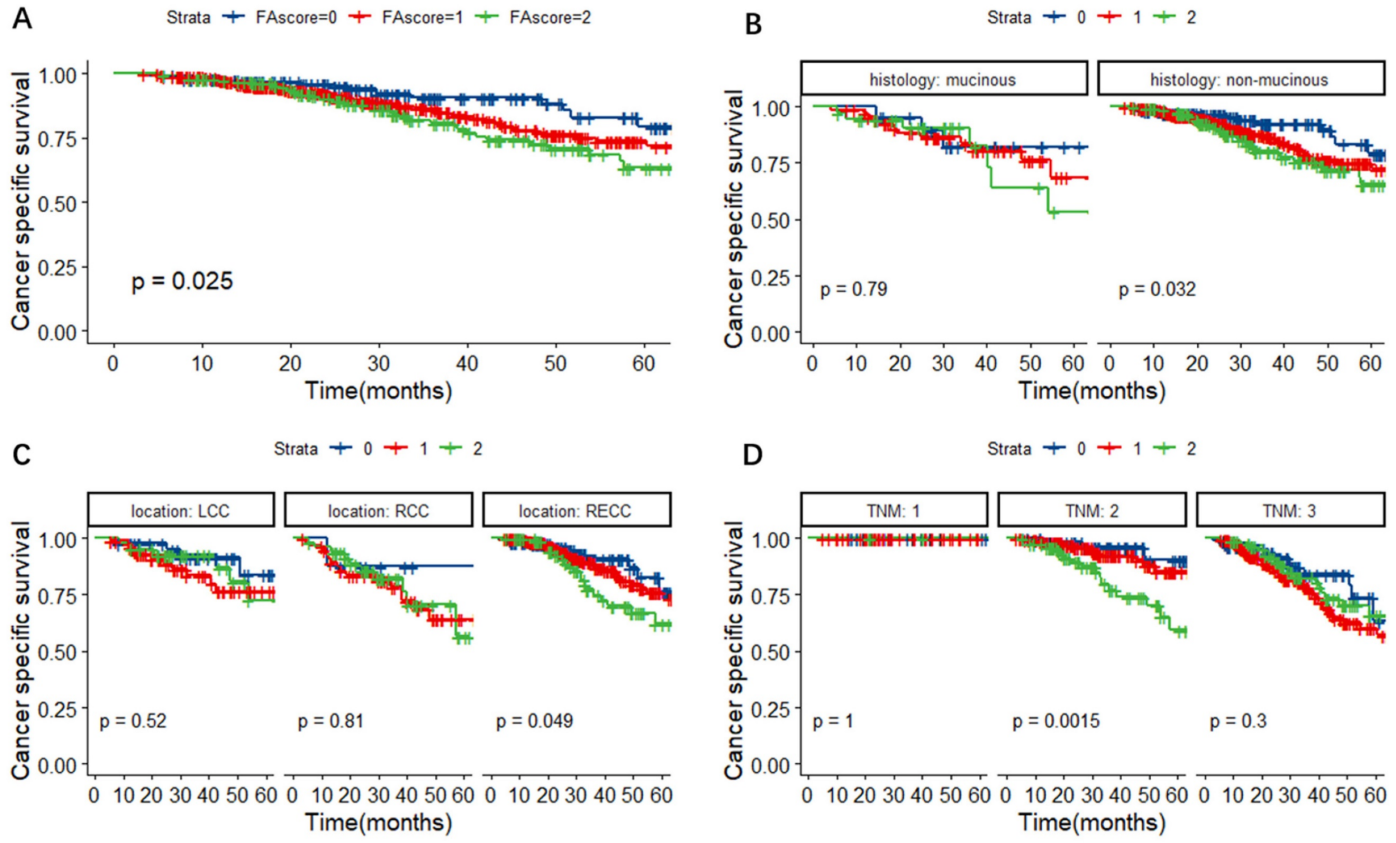
Kaplan-Meier survival curves for CSS according to the FA score (A) and subgroup analysis based on histological features (B) and primary tumor location (C) and TNM stage (D). **Abbreviations:** FA score, combined fibrinogen and albumin; CSS, cancer-specific survival.

In Figure 3A, we found that the FA score could predict the CSS of CRC patients (P = 0.025). Further subgroup analyses base on different histological types, primary tumor locations and TNM stages were performed. As shown in Figure 3B-D, patients with a higher FA score had a worse cancer-specific survival, especially in patients with non-mucinous adenocarcinoma (P = 0.032), RECC (P = 0.049) and TNM stage 2 tumors (P = 0.0015).

**Figure 4:**
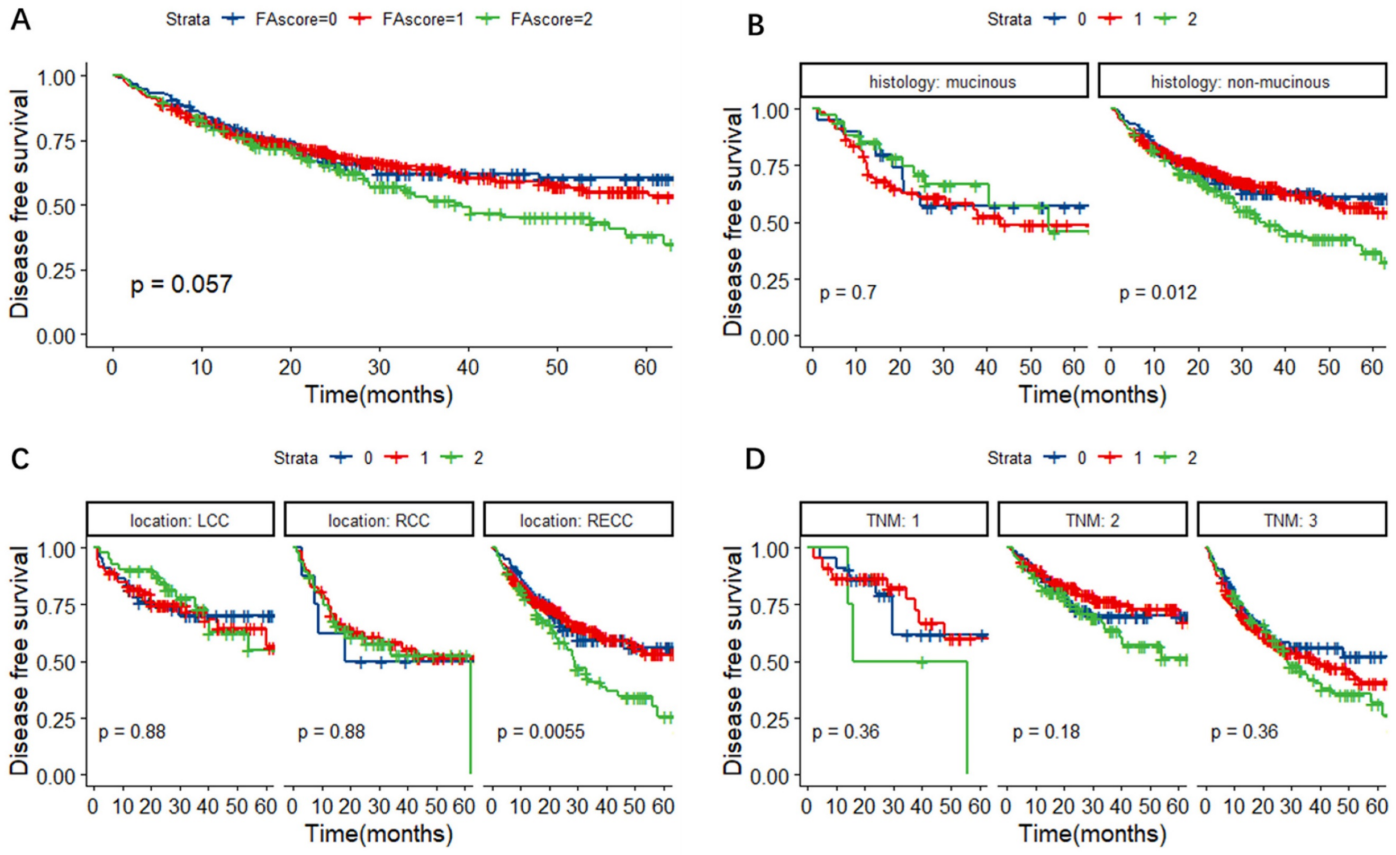
Kaplan-Meier survival curves for DFS according to the FA score (A) and subgroup analysis based on histological features (B) and primary tumor location (C) and TNM stage (D). **Abbreviations:** FA score, combined fibrinogen and albumin; DFS, disease-free survival.

In Figure 4A, it was observed that patients with a higher FA score might have a higher possibility of regression or metastasis, although the P value was slightly above 0.05 (P = 0.057). Further subgroup analyses revealed that the FA score could predict DFS in CRC patients with non-mucinous adenocarcinoma (P = 0.012) and RECC (P = 0.0055) (Figure 4B-D).

### FA score as a predictive factor for chemotherapeutic effectiveness in TNM stage III CRC patients

**Figure 5:**
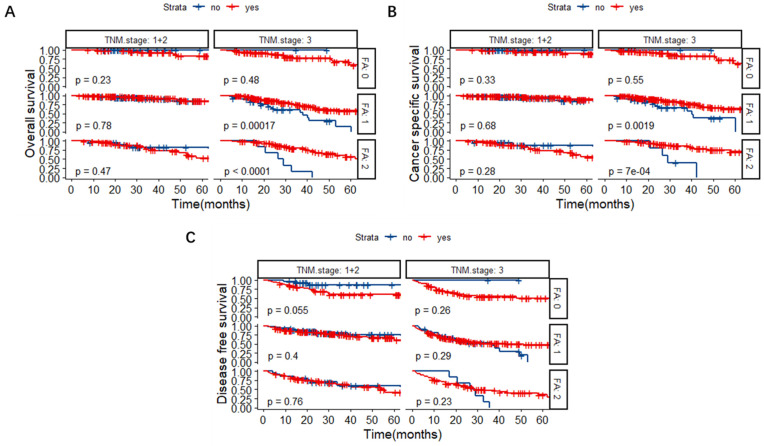
Kaplan-Meier survival curves based on chemotherapy or not in patients with different TNM stages and FA scores. (A) OS. (B) CSS. (C) DFS. **Abbreviations:** FA score, combined fibrinogen and albumin; OS, overall survival; CSS, cancer-specific survival; DFS, disease-free survival.

Patients were further divided into a chemotherapy group and a non-chemotherapy group based on whether they received post-operative chemotherapy or not. The Kaplan-Meier survival curves revealed that for TNM stage III CRC patients, the OS of FA score 1 and 2 groups could be lengthened significantly after the administration of chemotherapy (Figure 5A, P < 0.001). Similarly, chemotherapy could benefit the CSS in TNM stage III CRC patients with FA score 1 and 2 (Figure 5B, P = 0.0019 and P < 0.001, respectively).

## Discussion

Inflammation has been convincingly considered as one of the most important hallmarks of cancer 22. Growing evidence shows that systemic inflammation response participates in the initiation, development and progression of several malignancies 23-26. Therefore, a series of inflammation-based index systems, including SII 27, GPS 28, lymphocyte-monocyte-ratio (LMR) 29, 30, prognostic nutritional index (PNI) 31 and C-reactive protein/albumin ratio (CAR) 32, have been reported to predict the prognosis in a variety of tumors. The FA score, as a novel inflammation-based marker, was proposed and reported to have prognostic power in several types of cancers 16-20.

Plasma fibrinogen, which is well-known as a coagulation-related protein, is also considered to be involved in the angiogenesis, proliferation, migration and metastasis of tumor cells by directly binding to members of the vascular endothelial growth factor (VEGF), fibroblast growth factor (FGF), transforming growth factor β (TGF-β), and platelet derived growth factor (PDGF) families 33, thus regulating the inflammatory status and cancer progression 34. An increasing number of researches have shown that elevated level of plasma fibrinogen is a strong predictor of malignancy and is correlated with unfavorable outcomes in several solid tumors, including ovarian cancer and cholangiocarcinoma 35-37. Furthermore, hyperfibrinogen was associated with poor prognosis and advanced tumor stage in colorectal cancer 38.

Serum Albumin is commonly used as a parameter to reflect nutritional status. In tumor patients, the most important reason for the reduction of albumin concentration is not the synthesis disorder or accelerated transcapillary leakage rates, but the increasing degradation of albumin, secondary to systemic inflammatory responses to the host 39. It was demonstrated that proinflammatory cytokines released by tumor tissue and related inflammatory cells, such as IL-4 and IL-6, could affect the synthesis of albumin in hepatocytes, thus decreasing albumin levels 40. Therefore, albumin levels could also implicate inflammatory response. The clinical effect of hypoalbuminemia on colorectal cancer has also been investigated. In research of 431 patients with curative colorectal cancer, serum albumin level was identified as a reliable prognostic marker for survival 41.

Therefore, the FA score, as an integrated index based on the plasma fibrinogen and albumin, reflects the preoperative inflammatory responses of hosts to tumors and the alterations in the cancer microenvironment. We hypothesized that FA score could favor the cancer initiation, progression and metastasis. Thus, we performed the present study to assess the prognostic and predictive value of FA score in patients with resectable CRC.

In the present study, interesting associations between the FA score and clinicopathological characteristics were observed. FA score was associated with age, size, TNM stage and CEA level, supporting the above-mentioned hypothesis that the elevated FA score might favor tumor proliferation, invasion and metastasis. Univariate and multivariate analyses revealed that the FA score, T stage and N stage were independent risk factors for OS in resectable CRC patients. In addition, subgroup analyses based on the histological type and location revealed that the elevated FA score was correlated with poor OS, CSS and DFS in patients with colorectal non-mucinous adenocarcinoma and RECC. Subgroup analyses based on TNM stage revealed that the elevated FA score was correlated with poor OS and CSS in TNM II CRC patients. Furthermore, for TNM III CRC patients, elevated FA score may have better OS and CSS from the administration of chemotherapy.

To date, the TNM staging system is still the gold standard for doctors to predict the prognosis and select the treatment regimen for various types of malignancies. However, as the TNM staging system only reflects the pathological features of resected tumors after surgery, preoperative survival prediction and decision-making for further treatment was relatively difficult. Our findings noted the preoperative FA score as a novel clinical and prognostic biomarker for resectable CRC patients. Thus, as a simple, cheap, easy-acquired and convenient parameter in clinical practice, FA score may serve as a complementary to the TNM staging system to identify high-risk patients among patients with the same TNM stage. The FA score may help doctors to perform more careful surgeries and conduct more rigorous follow-up for these patients.

As we all know, the Liverpool score system which integrates the measurement of systemic inflammatory response and location of the primary tumor, is widely used for predicting the prognosis in patients with colorectal liver metastases 42. Compared with the Liverpool score system, the FA score system was easier to obtain and calculate, thus convenient for application. Additionally, the FA score could be used for predicting the prognosis and chemotherapy efficacy for CRC patients without liver metastasis. However, many other clinicopathological variables were not included and patients with liver metastasis were not applicable. In the future research, the FA score system could be investigated in colorectal liver metastases and thus might be effectively integrated into the Liverpool score system, which could be helpful for peri-operative management of high-risk patients.

To the best of our knowledge, this was the first research investigating the predictive value of FA score in prognosis and chemotherapeutic efficacy in patients with resected CRC. However, several limitations in the present study should be carefully considered. First of all, as a single-institution study in China, the total sample size was relatively small and the population diversity was relatively limited. Second, selection bias and collection mistakes could not be totally avoided due to the retrospective nature of the study. Third, the specific chemotherapy regimens were not investigated. Forth, the median follow-up time was shorter than 3 years, which was not sufficiently long. Fifth, the FA score at the time of pretreatment was evaluated, without taking into consideration of changes during treatment. Sixth, the AUC values for fibrinogen and albumin were slightly more than 0.5, which presented a relatively lower accuracy. Last but not least, only CRC patients who received curative surgery were included. Thus, the findings of this study might not be applicable for CRC patients in other countries or metastatic CRC. Therefore, a multi-center, large-scaled and prospective investigation is required to verify and update our conclusions in the future.

## Conclusion

In conclusion, the present study demonstrated the predictive value of the preoperative FA score in the prognosis and chemotherapeutic efficacy in patients with resected colorectal cancer. Patients with a higher FA score indicated higher risks of mortality. For TNM stage III CRC patients, chemotherapy might benefit the survival for patients with FA score 1 and 2. Therefore, FA score may serve as a complementary to the TNM staging system to identify high-risk patients who should receive more careful surgeries and post-operative chemotherapy.

## Figures and Tables

**Table 1:** Comparison of demographic and clinicopathological parameters between patients with different FA score.

Features	FA score			Overall	P*
	0 (n=165)	1 (n=399)	2 (n=171)	(N=735)	
Gender (%)					0.0325
Female	48 (29.09)	160 (40.10)	57 (33.33)	265 (36.05)	
Male	117 (70.91)	239 (59.90)	114 (66.67)	470 (63.95)	
Age (%)					<0.0001
≤60	125 (75.76)	227 (56.89)	86 (50.29)	438 (59.59)	
>60	40 (24.24)	172 (43.11)	85 (49.71)	297 (40.41)	
Smoking (%)					0.9195
No	134 (81.21)	319 (79.95)	136 (79.53)	589 (80.14)	
Yes	31 (18.79)	80 (20.05)	35 (20.47)	146 (19.86)	
Drinking (%)					0.4745
No	132 (80.00)	335 (83.96)	144 (84.21)	611 (83.13)	
Yes	33 (20.00)	64 (16.04)	27 (15.79)	124 (16.87)	
Location (%)					<0.0001
LCC	44 (26.67)	73 (18.30)	41 (23.98)	158 (21.50)	
RCC	8 (4.85)	58 (14.54)	48 (28.07)	114 (15.51)	
RECC	113 (68.48)	268 (67.17)	82 (47.95)	463 (62.99)	
Tumor size (%)					<0.0001
≤4cm	86 (52.12)	167 (41.85)	35 (20.47)	288 (39.18)	
>4cm	70 (42.42)	221 (55.39)	121 (70.76)	412 (56.05)	
Unknown	9 (5.45)	11 (2.76)	15 (8.77)	35 (4.76)	
Morphological type (%)					0.4163
Expansive	27 (16.36)	66 (16.54)	29 (16.96)	122 (16.60)	
Infiltrative	1 (0.61)	15 (3.76)	6 (3.51)	22 (2.99)	
Ulcerative	135 (81.82)	313 (78.45)	132 (77.19)	580 (78.91)	
Complex	2 (1.21)	5 (1.25)	4 (2.34)	11 (1.50)	
Histological type (%)					0.119
Non- mucinous	145 (87.88)	331 (82.96)	136 (79.53)	612 (83.27)	
Mucinous	20 (12.12)	68 (17.04)	35 (20.47)	123 (16.73)	
Differentiation (%)					0.0909
Well	3 (1.82)	14 (3.51)	3 (1.75)	20 (2.72)	
Moderate	128 (77.58)	290 (72.68)	112 (65.50)	530 (72.11)	
Poor	19 (11.52)	65 (16.29)	35 (20.47)	119 (16.19)	
Unknown	15 (9.09)	30 (7.52)	21 (12.28)	66 (8.98)	
Venous invasion (%)					0.5692
Negative	160 (96.97)	380 (95.24)	162 (94.74)	702 (95.51)	
Positive	5 (3.03)	19 (4.76)	9 (5.26)	33 (4.49)	
Perineural invasion (%)					0.2344
Negative	161 (97.58)	385 (96.49)	161 (94.15)	707 (96.19)	
Positive	4 (2.42)	14 (3.51)	10 (5.85)	28 (3.81)	
Tumor deposits (%)					0.7826
Absent	156 (94.55)	382 (95.74)	162 (94.74)	700 (95.24)	
Present	9 (5.45)	17 (4.26)	9 (5.26)	35 (4.76)	
T stage (%)					0.0905
1	4 (2.42)	12 (3.01)	2 (1.17)	18 (2.45)	
2	24 (14.55)	43 (10.78)	9 (5.26)	76 (10.34)	
3	46 (27.88)	100 (25.06)	50 (29.24)	196 (26.67)	
4	91 (55.15)	244 (61.15)	110 (64.33)	445 (60.54)	
N stage (%)					0.9943
0	79 (47.88)	197 (49.37)	83 (48.54)	359 (48.84)	
1	47 (28.48)	107 (26.82)	46 (26.90)	200 (27.21)	
2	39 (23.64)	95 (23.81)	42 (24.56)	176 (23.95)	
TNM stage (%)					0.0032
1	22 (13.33)	45 (11.28)	4 (2.34)	71 (9.66)	
2	58 (35.15)	151 (37.84)	79 (46.20)	288 (39.18)	
3	85 (51.52)	203 (50.88)	88 (51.46)	376 (51.16)	
Chemotherapy (%)					0.1448
No	30 (18.18)	90 (22.56)	27 (15.79)	147 (20.00)	
Yes	135 (81.82)	309 (77.44)	144 (84.21)	588 (80.00)	

**Notes:** *P-values were calculated by the χ2-test or the Fisher's exact test. The P-value for significance was <0.05. **Abbreviations:** LCC, left colon cancer; RCC, right colon cancer; RECC, rectal cancer; CEA, carcinoembryonic antigen; CA19-9, cancer antigen 19-9.

**Table 2:** Univariate and multivariate Cox analyses of OS in CRC patients.

	Univariate analysis			Multivariate analysis*		
	HR	P	95% CI	HR	P	95% CI
Gender (male/female)	1.07	0.692	0.76-1.5			
Age (≤60/>60)	0.71	0.038	0.51-0.98	0.70	0.036	0.50-0.98
Smoking	1.21	0.359	0.81-1.81			
Drinking	1.29	0.218	0.86-1.92			
Location						
RCC/LCC	1.52	0.118	0.9-2.55			
RECC/LCC	1.13	0.567	0.74-1.72			
Size (≤4/>4 cm)	0.89	0.507	0.62-1.26			
Morphological type						
expansive/complex	0.63	0.444	0.19-2.08			
infiltrative/complex	1.45	0.585	0.38-5.46			
ulcerative/complex	0.58	0.348	0.18-1.82			
Histology (muc/non)	1.28	0.231	0.86-1.91			
Differentiation						
moderate/well	0.72	0.471	0.29-1.77			
poor/well	1.00	0.998	0.38-2.59			
Venous invasion	2.14	0.009	1.21-3.79	1.26	0.460	0.68-2.33
Perineural invasion	2.45	0.01	1.24-4.83	1.45	0.325	0.69-3.02
Tumor deposits	2.01	0.02	1.12-3.64	1.42	0.252	0.78-2.59
T stage	1.58	0.001	1.21-2.07	1.35	0.028	1.03-1.77
N stage	1.75	<0.001	1.44-2.12	1.64	<0.001	1.34-2.02
Chemotherapy	0.82	0.333	0.55-1.23			
FA score	1.46	0.002	1.15-1.85	1.30	0.037	1.02-1.66
1/0	1.55	0.076	0.95-2.51	1.49	0.114	0.91-2.43
2/0	2.17	0.003	1.3-3.62	1.76	0.034	1.04-2.99
2/1	1.40	0.065	0.98-2.01	1.19	0.358	0.82-1.71

**Notes:** *Variables with a p-value <0.1 in univariate analysis were enrolled in a multivariate Cox proportional hazards regression model. **Abbreviations:** OS, overall survival; CRC, colorectal cancer; RCC, right colon cancer; LCC, left colon cancer; RECC, rectal cancer; muc, mucinous; non, non-mucinous; F, fibrinogen; A, albumin.

**Table 3:** Univariate and multivariate Cox analyses of CSS in CRC patients.

	Univariate analysis			Multivariate analysis*		
	HR	P	95% CI	HR	P	95% CI
Gender (male/female)	1.17	0.432	0.79-1.73			
Age (≤60/>60)	0.64	0.018	0.44-0.93	0.65	0.033	0.44-0.97
Smoking	1.16	0.532	0.73-1.85			
Drinking	1.31	0.245	0.83-2.08			
Location						
RCC/LCC	1.86	0.033	1.05-3.28	1.82	0.047	1.01-3.30
RECC/LCC	1.04	0.869	0.64-1.7	1.18	0.518	0.71-1.95
Size (≤4/>4 cm)	0.96	0.835	0.64-1.44			
Morphological type						
expansive/complex	0.47	0.229	0.14-1.61			
infiltrative/complex	1.27	0.729	0.33-4.92			
ulcerative/complex	0.43	0.15	0.13-1.36			
Histology (muc/non)	1.23	0.377	0.77-1.97			
Differentiation						
moderate/well	0.69	0.474	0.25-1.9			
poor/well	0.93	0.901	0.32-2.73			
Venous invasion	2.63	0.002	1.44-4.81	1.54	0.204	0.79-2.99
Perineural invasion	2.89	0.004	1.4-5.96	1.74	0.180	0.77-3.90
Tumor deposits	2.5	0.004	1.34-4.66	1.60	0.154	0.84-3.06
T stage	1.91	<0.001	1.36-2.67	1.60	0.008	1.13-2.28
N stage	1.73	<0.001	1.39-2.16	1.59	<0.001	1.24-2.02
Chemotherapy	0.85	0.506	0.53-1.36			
FA score	1.46	0.007	1.11-1.93	1.20	0.215	0.90-1.61
1/0	1.68	0.074	0.95-2.96	1.47	0.193	0.82-2.64
2/0	2.25	0.008	1.24-4.11	1.56	0.171	0.83-2.93
2/1	1.34	0.162	0.89-2.03	1.06	0.803	0.69-1.62

**Notes:** *Variables with a p-value <0.1 in univariate analysis were enrolled in a multivariate Cox proportional hazards regression model. **Abbreviations:** CSS, cancer specific survival; CRC, colorectal cancer; RCC, right colon cancer; LCC, left colon cancer; RECC, rectal cancer; muc, mucinous; non, non-mucinous; F, fibrinogen; A, albumin.

**Table 4:** Univariate and multivariate Cox analyses of DFS in CRC patients.

	Univariate analysis			Multivariate analysis*		
	HR	P	95% CI	HR	P	95% CI
Gender (male/female)	1.13	0.339	0.88-1.45			
Age (≤60/>60)	0.99	0.965	0.78-1.27			
Smoking	1.05	0.732	0.78-1.42			
Drinking	1.21	0.211	0.9-1.64			
Location						
RCC/LCC	1.75	0.007	1.17-2.62	1.79	0.005	1.19-2.70
RECC/LCC	1.51	0.014	1.09-2.09	1.48	0.023	1.05-2.07
Size (≤4/>4 cm)	0.94	0.634	0.73-1.21			
Morphological type						
expansive/complex	0.49	0.106	0.21-1.16			
infiltrative/complex	1.23	0.67	0.47-3.21			
ulcerative/complex	0.57	0.179	0.25-1.29			
Histology (muc/non)	1.08	0.626	0.79-1.47			
Differentiation						
moderate/well	1.46	0.402	0.6-3.55			
poor/well	2.03	0.132	0.81-5.07			
Venous invasion	1.88	0.008	1.18-3	1.15	0.569	0.70-1.89
Perineural invasion	3.1	<0.001	1.98-4.86	1.95	0.008	1.19-3.18
Tumor deposits	2.76	<0.001	1.83-4.17	1.93	0.003	1.25-2.98
T stage	1.22	0.019	1.03-1.45	1.03	0.712	0.86-1.24
N stage	1.63	<0.001	1.42-1.88	1.52	<0.001	1.30-1.77
Chemotherapy	1.37	0.054	1-1.89	1.05	0.788	0.75-1.46
FA score	1.21	0.033	1.02-1.44	1.12	0.202	0.94-1.34
1/0	1.06	0.702	0.78-1.45	1.05	0.771	0.76-1.44
2/0	1.43	0.041	1.01-2.01	1.25	0.220	0.88-1.78
2/1	1.34	0.035	1.02-1.77	1.19	0.229	0.90-1.58

**Notes:** *Variables with a p-value <0.1 in univariate analysis were enrolled in a multivariate Cox proportional hazards regression model. **Abbreviations:** DFS, disease free survival; CRC, colorectal cancer; RCC, right colon cancer; LCC, left colon cancer; RECC, rectal cancer; muc, mucinous; non, non-mucinous; F, fibrinogen; A, albumin.
